# Apgar Score for Newborn Dog Viability Assessment: Differences between English and French Bulldogs Born via Cesarean Section

**DOI:** 10.3390/ani13213318

**Published:** 2023-10-25

**Authors:** Maria Cristina Veronesi, Roberta Bucci, Monica Probo, Massimo Faustini, Jasmine Fusi

**Affiliations:** 1 Department of Veterinary Medicine and Animal Sciences, Università degli Studi di Milano, Via dell’Università 6, 26900 Lodi, Italy; maria.veronesi@unimi.it (M.C.V.); massimo.faustini@unimi.it (M.F.); jasmine.fusi@unimi.it (J.F.); 2 Department of Veterinary Medicine, University of Teramo, 64100 Teramo, Italy

**Keywords:** dog, newborn viability, Apgar score, English bulldog, French bulldog

## Abstract

**Simple Summary:**

After its first description, the use of AS for the evaluation of newborn dogs’ viability has become largely recognized in research and practical settings. Despite the undoubted usefulness, some aspects need to be better clarified. Among these is the possible need for breed-oriented AS modifications, especially when brachycephalic breeds are concerned. The present study aimed to clarify the possible differences in newborn viability between two brachycephalic breeds, often considered similar to each other, but characterized by marked differences. The results have proven that when viability assessed through the AS was concerned, significant differences between EB and FB puppies born via cesarean section were found. Therefore, although both are brachycephalic and bulldogs, EB and FB puppies are viable at birth to a different extent, especially from a neurologic point of view, with EB puppies being less reactive than FB newborns. For this reason, tailored breed-oriented care must be provided to puppies needing special assistance, even if the mortality rate at 7 days of age remains high in EB puppies and deserves a more detailed investigation.

**Abstract:**

Even if largely used in canine neonatology, some questions about the Apgar Score (AS) arose. Notably, considering the breed-oriented modifications of the score are needed, slight changes of the score were reported for English (EBs) and French bulldogs (FBs). The present study aimed to evaluate the possible differences between neonatal viability of the two breeds assessed by AS in puppies born via cesarean section. The results obtained from 99 puppies born alive and without malformations (EB = 47, FB = 52) showed significant (*p* < 0.001) differences in the median AS (seven in EB vs. eight in FB), with Grimace (*p* < 0.05 for sub-score 0 and *p* < 0.001 for sub-score 2) and Attitude (*p* < 0.05) being differently sub-scored between the two breeds. In the 89 alive puppies at 7 days of age, the same difference in the median AS was observed (*p* < 0.001), and only Grimace was differently sub-scored between the two breeds (*p* < 0.05 for sub-score 0 and *p* < 0.01 for sub-score 2). These results suggest that low Grimace could be an intrinsic characteristic of EB newborns, but it could negatively affect the neonatal adaptation process of EBs, providing an indication for focused neonatal assistance. Neonatal mortality at 7 days of age was higher in EBs than in FBs (17 vs. 3.8%, respectively), which deserves further investigations. The study provides further evidence that breed-oriented ASs are needed for a better evaluation and assistance of purebred puppies at birth.

## 1. Introduction

Albeit underestimated until today, canine neonatology is a topic of interest for veterinarians involved in the reproductive medicine of pets. Despite the increasingly professional and targeted approach aimed at decreasing the number of non-surviving puppies in dogs, the percentages of perinatal mortality, considered as the sum of stillborn and neonatal deaths in this species, can reach values of up to 40% still today [1], representing an ethical, economic, and professional issue for both veterinarians and breeders. Birth is a crucial process for the newborn, representing a sudden passage from the protected intrauterine to the unprotected external environment. Indeed, as dogs are a polytocous species, the expulsion phase can last several hours, also depending on the litter size. This can predispose the fetuses to hypoxia, impairing their viability [2,3,4,5] and being a main cause of mortality around birth [5]. Moreover, some breeds are considered to be at a high risk for dystocia, also responsible for impairing newborn viability [6]. Two factors can contribute to limiting perinatal losses: the correct management of parturition [6,7] and the prompt recognition of newborn puppies requiring additional assistance [8]. Regarding the former, the aim is to avoid any cause of dystocia, limiting the life-threatening conditions for the newborns. In breeds predisposed to dystocia, like English Bulldogs (EBs) and French Bulldogs (FBs), an elective cesarean section can be planned, aimed at the best well-being, and preventing possible negative consequences to newborns and mothers [8,9,10,11].

The latter, on the other hand, is pivotal for the newborn outcome.

Neonatal assessment of viability soon after birth is indeed a necessary pre-requisite for optimal newborn resuscitation and assistance, allowing for the best neonatal survival [9,12,13,14].

Newborn viability can be influenced by body temperature (BT), with hypothermia reported as one of the main causes of neonatal mortality [15,16], so the measurement of BT after birth has been suggested by several studies [13,17,18]. However, all these studies demonstrated that rectal temperature was not significant as a predictor of neonatal mortality. Newborn dog viability can be evaluated by assessing the presence and/or strengths of some vital behaviors/reflexes, in some cases assessed as absent or present [13] and in others being scored [17].

The Apgar score is one of the most used methods for assessing newborn viability in humans and animals. More than a decade ago, an Apgar Score (AS) was introduced by Veronesi and colleagues [13] to evaluate canine newborns’ viability at birth, allowing for the classification of puppies in normal viable, less viable, and critical newborns [13], providing a useful tool for short-term survival prognosis. Given its ease and usefulness, since its first description, AS has been extensively used in studies involving different canine breeds, even if some authors made some changes, mainly related to the AS cut-off values for the viability classification [9,10]. In 2014, Batista and co-authors [9] suggested slight modifications based on breed-oriented ASs for specific evaluations of English and French bulldogs. In comparison to the AS model proposed by Veronesi et al. [13], the main change proposed by Batista et al. [9] regarded the parameter Pulse, with a different heart rate range, for a better evaluation of viability in these breeds. After that study, other researchers focused on the neonatal evaluation of brachycephalic puppies at birth; some used the AS proposed by Veronesi et al. [13] and others used the one proposed by Batista et al. [9], so currently, both models can be found in the literature for the viability assessment at birth in brachycephalic newborns.

However, the peculiarities of EB and FB puppies remain a topic of interest in canine neonatology. It was reported that both EB and FB newborns are more prone to neonatal death in the first hours of life, thus requiring more individual assistance and care than other breeds [19,20,21]. Moreover, French Bulldog newborns, were reported to need additional resuscitation procedures in more than 50% of the puppies at birth [21], but similar data about EB are lacking.

Even if EBs and FBs are often considered similar breeds, as they are both brachycephalic and bulldog dogs, they are morphologically distinct, with different body sizes and weights. In relation to newborn viability at birth, practitioners have the belief that EB puppies are usually less viable than FB puppies, needing more assistance immediately after birth. Recently, [10] reported a refining of the viability classification on a considerable number of newborn dogs, and suggested a different viability classification of EBs and FBs based on their diverse body size.

Because of the above-mentioned reasons, the aim of the present study was to go into detail about the AS evaluation of EB and FB newborn puppies. Possible differences between the two breeds were investigated regarding (1) the perinatal mortality; (2) the total AS, the viability classification, and the neonatal reflexes in the puppies born alive and without malformations; (3) sub-score of each one of the five parameters used to assess the AS in the puppies born alive and without malformations; (4) the total AS, the viability classification and the neonatal reflexes in the puppies alive at 7 days of age; (5) sub-score of each one of the five parameters used to assess the AS in the alive puppies at 7 days of age; (6) total AS and sub-score of each one of the five parameters used to assess the AS between male and female newborns among EBs and FBs.

## 2. Materials and Methods

### 2.1. Ethics

The study was conducted following ethical guidelines provided by the animal welfare committee, and all the procedures were carried out according to the Italian legislation about animal care (DL 116, 27 January 1992) and the European Guidelines on Animal Welfare (Directive 2010/63/EU). A written informed consent was signed by each owner to submit the bitches to elective cesarean section, to allow all the needed clinical procedures on mothers and newborns and use the clinical records for research purposes.

### 2.2. Animals

The study was performed on newborn puppies from 11 EB and 10 FB bitches, all submitted to elective cesarean section because of the high risk of dystocia reported for both breeds [6]. All the enrolled bitches were healthy, regularly submitted to routine vaccination and deworming protocols, with a body condition score of 5/9 or 6/9 at mating. The bitches were fed a commercial diet, with constant modifications throughout pregnancy to supply nutritional and metabolic requirements, also in consideration of the number of carried fetuses. Pregnancy was monitored from the time of mating until parturition. Other than plasma progesterone concentration measurement at mating, elective cesarean sections were planned based on fetal biometry [22,23,24]. Furthermore, case-by-case plasma progesterone concentration monitoring in the last days of pregnancy allowed for the most precise timing in scheduling the births, as previously reported [8,25]. In all the cases, during the last week of pregnancy, case-by-case monitoring of the mothers and fetal viability and well-being was performed via clinical and ultrasonographic examinations.

### 2.3. Anesthetic and Surgical Procedures

In all the cases the same anesthetic protocol previously reported [25], was applied to minimize the possible side effects on mothers and newborns. Briefly, premedication was performed with atropine (0.02 mg/kg IM) and metoclopramide (0.2 mg/kg SC), then cefazolin was administered (25 mg/kg IV), mask oxygenation was provided and, lastly, induction was performed with propofol (4–6 mg/kg IV). Lidocaine (2 mg/kg) was injected at the site of the surgical incision. Anesthesia was then maintained using isoflurane in oxygen. The elective cesarean sections were performed with a ventral midline laparotomy technique. Immediately after all the fetuses were extracted, tramadol (3 mg/kg IV) and oxytocin (0.15 IU/kg IM) injections were administered to the bitches. As soon as each newborn was extracted from the uterus, two expert neonatologists (MCV and FJ) provided an assessment of and assistance to the newborn. The surgery time, considered the time elapsing between the induction and extraction of the last puppy, was recorded.

### 2.4. Newborn Puppy Assessment

After extraction, each puppy was submitted for a single evaluation of viability using the AS, as reported by Veronesi et al. [13].

Briefly, each puppy born alive was assessed for membrane mucus color (Appearance), heart rate (Pulse), irritability reflex (Grimace), movement (Attitude), and respiratory rate/efforts (Respiration). A sub-score from 0 to 2 was assigned to each parameter (0 as the worst and 2 as the optimal), for a final score ranging between 0 and 10 [13]. The final score assigned to each newborn puppy allowed for classification into one of the three classes of newborn viability in relation to breed body size, as suggested by Veronesi et al. [10]. For small-sized breeds such as FBs, the scores are as follows: no distress for an AS of 5–10, moderate distress for an AS of 4, and severe distress for an AS of 0–3. For large-sized breeds such as EBs, the scores are as follows: no distress for an AS of 8–10, moderate distress for an AS of 4–7, and severe distress for an AS of 0–3 [10].

Immediately after Apgar scoring, because newborn viability can also influence some vital behaviors/reflexes, newborn puppies were also evaluated for some reflexes important for viability, such as searching for the mammary gland (MG), suckling (SK), and swallowing (SW) reflexes [13], each scored as 1 when absent, 2 when weak, and 3 when strong. The search for the mammary gland was assessed by positioning the muzzle of each puppy within a circle created by the thumb and the forefinger of the examiner, and assessing the triggering of the search, and suckling by inserting the tip of a finger in the mouth and assessing the force of the suctioning of the puppy. The swallowing reflex was assessed by observing the ability and strengths of swallowing after the administration of a drop of energetic solution to the puppy (Energy Booster, Ozopet, Mantua, Italy). The Apgar score was assessed at 5 min after birth, when the first stabilization (drying, initial clearing of the airways, thorax rubbing, umbilical cord clamping) of the newborn was already provided to have a more real indication of the newborn vitality. After these first cares (provided to all the newborns), and checking for the absence of malformations, additional assistance was provided to each newborn, depending on their class of vitality. No distressed newborns were forwarded to routine neonatal management including heating, birthweight, and body temperature measurement, and they were put in an incubator until their mothers recovered from anesthesia. Moderately distressed newborns received a different degree of assistance/resuscitation according to the degree of distress. This tailored assistance starts with manual additional breathing stimulation via tactile stimulations and thorax rubbing, and oxygen administration through a face mask when needed. Severely distressed newborns received oxygen through a face mask and lateral chest compression over the cardiac area; when no heartbeat was detected, they underwent trans-thoracic cardiac compression and epinephrine (0.01 mg/kg Adrenalina Salf^®^, Salf S.p.A. Laboratorio Farmacologico, Bergamo, Italy) [13,26]. Newborn rectal body temperature (BT) was measured at the end of routine care or assistance using a digital thermometer, characterized by a measuring range of 32–42.9 °C and a precision of ±0.1–0.2 °C (Torm 10 smt-403 s, Hangzhou Sejoy Electronics & Instruments Co., Hangzhou, China).

Newborn sex and birthweight (BW) were recorded, the latter using an electronic scale (calibrated at 1 g increment) before the newborn started to nurse. At the end of the surgery and after the complete awakening of the bitches, the display of normal maternal behavior was checked before the medical discharging of the mothers and litters. The presence of normal mammary secretions was also tested; if maternal milk was not available, artificial milk with specific canine newborn formula was administered (Puppy Pro-Tech, Royal Canin, Gallargues-le-Montueux, France) every 2 h at the dose of 3 mL/100 g of BW.

Regarding the newborns, the following data were recorded: the total number of puppies born, the number of stillborn, the number of puppies with severe malformations requiring humane euthanasia, the number of puppies born alive and without malformations, and the number of newborn puppies alive at 7 days of age.

### 2.5. Statistics

After normality data distribution assessment employing the Anderson–Darling test, the non-parametric Kruskal–Wallis test was used to assess the possible differences between EBs and FBs for stillborn/euthanized puppies and puppies dead at 7 days of age, for maternal age and parity, surgery time, litter size, AS, viability classification, BT, BW, MG, SK, and SW in the puppies born alive and without malformations, and in the puppies alive at 7 days of age. Afterward, contingency tables were used to show the number and percentages of newborns distributed within each AS parameter sub-scoring in EBs and FBs. Then, the chi-square test was used to assess the possible differences in each AS parameter sub-score between EBs and FBs according to (1) the total puppies born alive and without malformations; (2) the puppies alive at 7 d of age; (3) the sexes of newborn among EBs and FBs. The Spearman test was used to assess possible correlations between AS and neonatal reflexes, and between viability classification and neonatal reflexes. Significance was set for *p* < 0.05 (Jamovi ver.2.4.1.).

## 3. Results

### 3.1. Newborn Assessment and Survival

From the 11 EB and 10 FB bitches enrolled, a total of 103 puppies were born, 49 EBs (24 males and 25 females) and 54 FBs (23 males and 31 females).

Of the 103 newborn puppies born, 2 FBs (both females) were stillborn, and 2 EBs (both females) were affected by severe cleft palate and therefore euthanized, and at 7 days of age, an additional 2 FBs (both females) and 8 EBs (5 females and 3 males) died; 5 out of 8 EBs died in the first 24 h of age. Data about stillborn, euthanized puppies, and puppies dead at 7 days of age, in EBs and FBs, are reported in Table 1.

Therefore, data about viability were collected from a total of 99 (47 EBs, 52 FBs) puppies born alive and without malformations.

### 3.2. Apgar Scores, Viability Classes, and Neonatal Reflexes according to the Viability Classification in the 99 Puppies Born Alive and without Malformations among EBs and FBs

**Figure 1:**
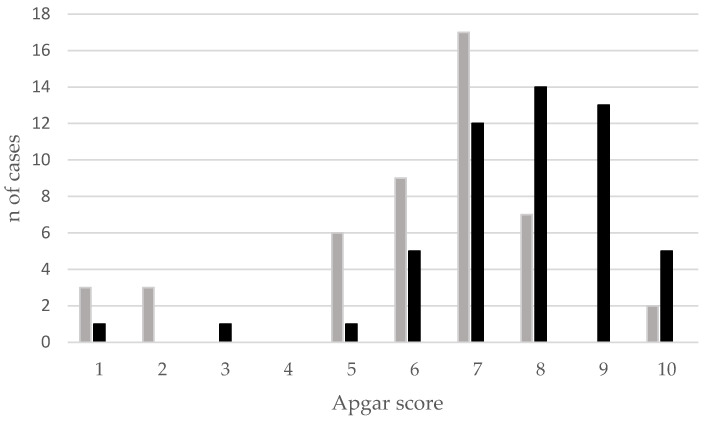
Distribution of the Apgar scores (number of cases) among the 99 puppies born alive and without malformations (EB, grey bar; FB, black bar).

The distribution of the Apgar scores (number of cases) in 99 newborn puppies, born alive and without malformations among EBs and FBs, is reported in Figure 1.

The distribution of the viability classes among the 99 EB and FB puppies born alive and without malformations is reported in Table 2.

Data about MG, SK, and SW for the 99 EB and FB puppies born alive and without malformations are reported in Table 3.

The Spearman correlation test showed significant positive correlations between the AS and MG (R = 0.45, *p* < 0.001), AS and SK (R0.38, *p* < 0.001), and AS and SW (R = 0.38, *p* < 0.001). Likely, a significant positive correlation was found between the viability classification and all the reflexes (MG, R = 0.54, *p* < 0.001; SK, R = 0.49, *p* < 0.001; SW, R = 0.49, *p* < 0.001).

### 3.3. Maternal Age, Parity, Surgery Time, Litter Size, AS, BW, and BT of the 99 EB and FB Puppies Born Alive and without Malformations

Data about maternal age, parity, surgery time, litter size, AS, BW, BT, MG, SK, and SW of the 99 (EB = 47; FB = 52) puppies born alive and without malformations are reported in Table 4.

### 3.4. Differences in Each Apgar Parameter Sub-Score between the 99 EB and FB Puppies Born Alive and without Malformations

The statistical analysis showed that neither Appearance nor Pulse and Respiration had significantly different sub-scores between EBs and FBs.

Contrastingly, Grimace (Table 5) and Attitude (Table 6) resulted in significantly more frequent higher sub-scores for FB puppies than EBs.

### 3.5. Apgar Score, Viability Classes, and Neonatal Reflexes between the 89 EB and FB Puppies Alive at 7 Days

**Figure 2:**
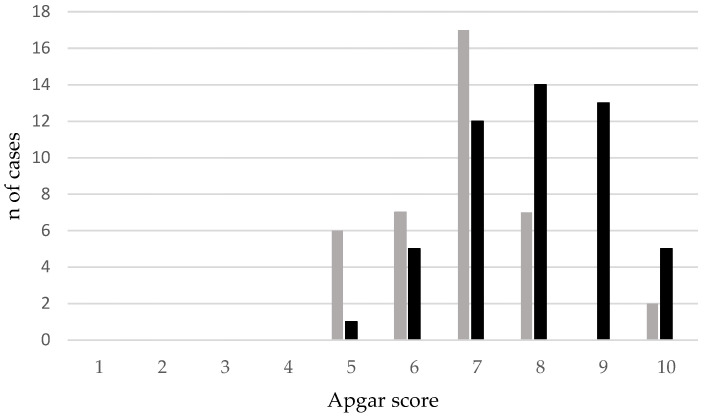
Distribution of the Apgar scores (number of cases) among the 89 puppies alive at 7 days of age (EB, grey bar; FB, black bar).

The distribution of the Apgar scores among the 89 puppies alive at 7 days of age is reported in Figure 2.

The distribution of the 89 EB and FB puppies alive at 7 days of age into viability classes is reported in Table 7.

Regarding the distribution of dead puppies, the two FB puppies that died within 7 days of age were classified as severely distressed newborns, while among the eight EB dead puppies, six were classified as severely distressed and two as not distressed newborns.

The possible differences in MG, SK, and SW between the 89 EB and FB puppies alive at 7 days of age are reported in Table 8.

The Spearman correlation test showed significant positive correlations between the AS and MG (R = 0.45, *p* < 0.001), AS and SK (R0.38, *p* < 0.001), and AS and SW (R = 0.38, *p* < 0.001). It is likely that a significant positive correlation was found between the viability classification and all the reflexes (MG, R = 0.54, *p* < 0.001; SK, R = 0.49, *p* < 0.001; SW, R = 0.49, *p* < 0.001).

### 3.6. Maternal Age, Parity, Surgery Time, Litter Size, AS, BW, and BT of the 89 EB and FB Puppies Alive at 7 Days of Age

Data about maternal age, parity, surgery time, litter size, AS, BW, and BT between the 89 EB and FB puppies alive at 7 d of age are reported in Table 9.

### 3.7. Differences in Each Apgar Parameter Sub-Score between the 89 EB and FB Puppies Alive at 7 Days of Age

Because of the different ASs between the two breeds, the possible differences in each AS parameter sub-score between the 89 EB and FB puppies alive at 7 d of age were assessed. The statistical analysis showed significant differences only for Grimace (Table 10).

### 3.8. Differences in the Total AS and Each Apgar Parameter Sub-Score between Male and Female Newborns EBs and FBs

The statistical analysis showed the absence of a significant difference in the total AS between male and female puppies within each breed for the 99 puppies born alive and without malformations (EB = 24 males and 23 females; FB = 23 males and 29 females), and the 89 puppies alive at 7 days of age (EB = 21 males and 18 females; FB = 23 males and 27 females). However, a significant difference in Attitude sub-scoring between males and females was found only for EB (*p* < 0.05) when the 99 puppies born alive and without malformations were considered. However, the chi-square test failed to evidence which sub-score was different between the males and females. From a descriptive point of view, females were scored as “0” (12 females vs. 6 males) or “2” (3 females vs. 0 males) more frequently, while males were scored as “1” (17 males vs. 9 females) more frequently.

## 4. Discussion

After its first description [13], the use of AS for newborn dogs’ viability evaluation has become largely recognized within research and practical settings. The present study confirmed the usefulness of the AS for viability assessment in newborn dogs, and confirmed the positive correlations between the AS and viability classification, with vital reflexes such as MG, SK, and SW, as previously reported [13].

Despite its undoubted usefulness, some aspects of the AS are yet to be better clarified. Among these is the possible need for breed-oriented AS modifications, especially when brachycephalic breeds are concerned. For this reason, the Apgar score refined according to the breed body size, as proposed by [10], was used.

An additional factor that could influence the AS is the timing of its measurement. In agreement with previous studies, the AS was assessed at 5 min after birth [8,9,10,13,27] because in newborn puppies, an adequate recovery occurs within 5 min after the initial depression due to the transition from the intra- to the extrauterine life [28].

The present study aimed to clarify the possible differences in newborn viability assessed through the AS between the two brachycephalic breeds, often considered similar to each other, but characterized by marked differences. Because newborn viability could be influenced by many factors, it is of note that, in the present study, no differences between EBs and FBs were found regarding maternal age and parity, surgery time, and anesthetic protocols. Similarly, neonatal assessment, resuscitation, and assistance were always provided by the same neonatologists, with similar clinical experience and skills.

The viability assessment through the AS showed significant differences between EB and FB puppies born via cesarean section. Interestingly, the median AS measured for all the puppies born alive and without malformation (8 vs. 7 FBs and EBs, respectively), and the median AS measured for puppies alive at 7 days of age (8 vs. 7 FBs and EBs, respectively) were different between the two breeds. It should be noted that according to the redefined viability classes recently proposed by Veronesi and co-authors [10] in puppies born via cesarean section, an AS = 8 for FBs is classified as no distress, while an AS = 7 for EBs must be considered moderate distress. Also, the proportion of EB and FB puppies distributed into the viability classes was different between the two breeds. Of the 99 puppies born alive and without malformations, more EBs than FBs were classified as moderately distress newborns, whilst of the puppies alive at 7 days of age, a significantly higher proportion of FBs than EBs was classified as the not distress. Thus, these results highlighted that EB puppies should be considered “less viable” than FB puppies at birth, needing more attention during the immediate postnatal assistance, as practically experienced by veterinary neonatologists.

When analyzing each AS parameter assessed in detail, it was evidenced that, when all newborns born alive and without malformations were considered, EB and FB puppies differed for the parameters Grimace and Attitude, with a higher proportion of EB puppies sub-scored 0 for Grimace and Attitude, and a higher proportion of FB puppies sub-scored 2 only for Grimace. Considering the puppies alive at 7 days of age, EBs and FBs differed only for Grimace, with a higher proportion of EB puppies sub-scored 0, and a higher proportion of FBs sub-scored 2. Interestingly, no differences were found for the parameters Appearance and Pulse, related to the heart and respiratory efficiency. These results are very remarkable because they highlight that the “lower reactivity” of EB newborns could be considered one of the many factors that could influence neonatal adaptation in this canine breed. Grimace, in fact, denotes the ability of a newborn to react to a disturbing stimulus, while Attitude refers to the strength of the body movement, both being coordinated by a normally developed and efficient neuromuscular system of the newborn [29]. Newborn reactivity and adequate movement are very important because they allow for and reflect the ability of the newborn to perform vital actions such as reaching the mammary gland and sucking and swallowing efficiently. Therefore, it is possible to speculate that insufficient Grimace and Attitude could negatively influence neonatal adaptation. However, the different Grimace between EBs and FBs, also observed in EBs alive at 7 days of age, seems to suggest that this aspect is probably almost physiologic in EBs, with no great consequences on survival. On the other hand, if low Grimace seems to be EB newborns’ peculiarity, the neonatologist should probably provide these newborns additional and focused stimulation immediately after birth, maybe through more specific tactile stimulation. It could be very interesting to understand the underlying causes for this “lower reactivity”, maybe scheduling a neurologic examination for the newborn puppy based on what is described in newborn infants [30]. Although the anesthetic protocol used in this study was the same for both breeds, some characteristics of EBs, such as different body mass index and/or the presence of adipose tissue, could have influenced the absorption of the propofol used for induction, and, in turn, affected the newborn reactivity. Body temperature, one factor that could have affected viability in general, and specifically newborn reactivity, was within the normal range reported for newborn dogs by Mila et al. [18] in all puppies, and without differences between EBs and FBs, so it is possible to suppose that, in the present study, BT did not play a role in the viability and reactivity of puppies. In the study by [30], neonatal primitive reflexes such as suckling and swallowing were considered useful for neurologic examination in newborn infants, providing information about the brainstem and cortical function. The absence of primitive reflexes in newborn babies may suggest a general depression at the central or peripheral nervous system motor function level. In the present study, the absence of MG, SK, and SW was associated with severe distress and 100% death in newborn puppies for both EBs and FBs. When analyzed according to the breed, significantly stronger MG, SK, and SW were found in FB than in EB puppies born alive and without malformations, while only MG remained stronger in FB than EB puppies alive at 7 days of age. These findings seem to suggest that the weaker SK and SW reflexes observed in EBs could have a role in neonatal mortality. Moreover, the weaker MG observed in EB than FB newborns surviving at 7 days of age seems to support the hypothesis that EB newborns could probably benefit from focused and tailored assistance to reach the mammary gland immediately after birth when SK and SW are present.

When sex-related differences were assessed, only Attitude within EBs resulted in significant differences (*p* < 0.05), but the statistical analysis failed to detect which sub-score was different between the males and females. From a descriptive standpoint, a higher proportion of females were sub-scored 0 or 2, while males were sub-scored 1 more frequently. A previous study in which a possible sex-related effect on the AS was investigated reported no differences in the AS between male and female newborn Chihuahua puppies [8] born via cesarean section. In humans, [31] reported a lower AS measured in male than female babies at 1 and 5 min after birth, suggesting a possible influence of sympatho-adrenergic system activity. However, if any, the sex-related effect on the AS in humans and animals needs to be better clarified.

Finally, regarding perinatal losses, an overall 14% was observed in the present study, with similar stillborn/euthanized puppies between EBs and FBs, but with a significant difference in neonatal mortality rate at 7 days of age between the two breeds (17 vs. 3.8% EBs and FBs, respectively). Compared with data about brachycephalic breeds previously reported, when cesarean section is considered, 14% of perinatal mortality aligns with the 11.6–14.9% reported by Widooghe et al. [20] and Batista et al. [9], but was higher than the 5.2% reported by Adams et al. [32]. Different clinical aspects (such as type of birth, time of observation after birth, etc.), however, could impact the different perinatal mortality rates found. Furthermore, sometimes studies do not discern among the puppies born dead, those euthanized because of malformations, and neonatal mortality of the puppies born alive and normal. The particularly high mortality rate in EBs, however, suggests that a broader investigation is needed to understand the underlying causes of neonatal mortality in this breed. Six out of eight dead EB puppies were Apgar-scored <3, as well as all four dead FB puppies. These results, on one hand, once more confirm the negative prognostic role of low AS on short-term survival, as previously reported [8,9,18]. On the other hand, puppies with a low AS probably suffer from multi-organ insufficiency or, at least, inefficiency of vital organs and/or systems, often making neonatal resuscitation vain. In fact, in the present study, 7/7 newborns with AS < 3 died despite the initial response to neonatal resuscitation, very similar to the approximate 93% mortality within the first 24 h of age reported by Batista et al. in brachycephalic puppies born via cesarean section [9].

## 5. Conclusions

In conclusion, the results of the present study showed that although both are brachycephalic and bulldogs, EB and FB puppies born via cesarean section are differently viable at birth, especially from a neurologic point of view, with EB puppies less reactive than FB newborns. Therefore, tailored breed-oriented care must be provided to puppies needing special assistance, even if the mortality rate at 7 days of age remains high in EB puppies, and deserves a more detailed investigation.

## Figures and Tables

**Table 1:** Stillborn and euthanized puppies, and puppies dead at 7 days of age, expressed as the number and (%) of EBs and FBs.

	Stillborn/Euthanized Puppies n (%)	Puppies Dead at 7 Days n (%)
EB (n = 49)	2/49 (4.1)	8/47 (17) ^a^
FB (n = 54)	2/54 (3.7)	2/52 (3.8) ^b^
TOTAL (n = 103)	4/103 (3.9)	10/99 (10.1)

^a,b^ denotes significant differences within columns, with *p* < 0.01.

**Table 2:** Distribution of the viability classes expressed as the number and (%) of the 99 EB and FB puppies born alive and without malformations.

	Severe Distress n (%)	Moderate Distress n (%)	No Distress n (%)
EB (n = 47)	6 (12.8)	6 (12.8) ^a^	35 (74.4)
FB (n = 52)	2 (3.8)	0 (0) ^b^	50 (96.2)
TOTAL (n = 99)	8 (8.1)	6 (6)	85 (85.9)

^a,b^ denotes significant differences within the columns, with *p* < 0.01.

**Table 3:** Data expressed as the median (min–max) of the MG, SK, and SW for the 99 EB and FB newborn puppies born alive and without malformations.

	EB	FB
MG	2 (1–3) ^a^	3 (1–3) ^b^
SK	2 (1–3) ^c^	3 (1–3) ^d^
SW	2 (1–3) ^c^	3 (1–3) ^d^

^a,b^ denotes significant differences within the rows, with *p* < 0.05; ^c,d^ denotes significant differences within rows, with *p* < 0.001.

**Table 4:** Data expressed as the median (min–max) of the maternal age, parity, surgery time, litter size, AS, BW, BT of the 99 (EB = 47; FB = 52) puppies born alive and without malformations. The surgery time was considered the time elapsing between the induction and extraction of the last puppy.

	EB	FB
MATERNAL AGE (years)	3 (2–5)	2 (2–6)
PARITY (n)	1 (1–3)	1 (1–3)
SURGERY TIME (min)	24 (15–30)	23 (17–26)
LITTER SIZE (n)	5 (2–10) ^a^	6 (3–6) ^b^
AS	7 (1–10) ^c^	8 (1–10) ^d^
BW (g)	350 (217–490) ^c^	270 (120–370) ^d^
BT (°C)	33.3 (32.1–34.7)	33.5 (32.1–34.5)

^a,b^ denotes significant differences within rows, with *p* < 0.05; ^c,d^ denotes significant differences within rows, with *p* < 0.001.

**Table 5:** Distribution of the sub-scores for Grimace expressed as the number and (%), assessed at 5 min after birth, for EB and FB puppies.

	Grimace		
	Sub-Score 0 n (%)	Sub-Score 1 n (%)	Sub-Score 2 n (%)
EB (n = 47)	17 (36.2) ^a^	23 (48.9)	7 (14.9) ^c^
FB (n = 52)	3 (5.7) ^b^	20 (38.5)	29 (55.8) ^d^
TOTAL (n = 99)	20 (20.2)	43 (43.4)	36 (36.4)

^a,b^ denotes significant differences within columns, with *p* < 0.05; ^c,d^ denotes significant differences within columns, with *p* < 0.001.

**Table 6:** Distribution of Attitude sub-scores expressed as the number and (%), assessed at 5 min after birth, for EB and FB puppies.

	Attitude		
	Sub-Score 0 n (%)	Sub-Score 1 n (%)	Sub-Score 2 n (%)
EB (n = 47)	18 (38.3) ^a^	26 (55.3)	3 (6.4)
FB (n = 52)	6 (11.5) ^b^	35 (67.3)	11 (21.2)
TOTAL (n = 99)	24 (24.2)	61 (61.7)	14 (14.1)

^a,b^ denotes significant differences within columns, with *p* < 0.05.

**Table 7:** Distribution into viability classes expressed as the number and (%)of the 89 EB and FB puppies alive at 7 days of age (the severe-distress class was deleted because none of the puppies in this class were alive at 7 days of age).

	Moderate Distress n (%)	No Distress n (%)
EB (n = 39)	6 (15.4)	33 (84.6) ^a^
FB (n = 50)	0 (0)	50 (100) ^b^
TOTAL (n = 89)	6 (6.7)	83 (93.3)

^a,b^ denotes significant differences within columns, with *p* < 0.05.

**Table 8:** Data expressed as the median (min–max) of the MG, SK, and SW for the 89 EB and FB puppies alive at 7 days of age.

	EB	FB
MG	2 (1–3) ^a^	3 (1–3) ^b^
SK	3 (2–3)	3 (2–3)
SW	3 (2–3)	3 (2–3)

^a,b^ denotes significant differences within rows, with *p* < 0.05.

**Table 9:** Data expressed as the median (min–max) of maternal age, parity, surgery time, litter size, AS, BW, and BT of the 89 puppies alive at 7 d of age.

	EB	FB
MATERNAL AGE (years)	3 (2–5)	2 (2–6)
PARITY (n)	1 (1–3)	1 (1–3)
SURGERY TIME (min)	24 (15–30)	23 (17–26)
LITTER SIZE (n)	5 (2–10) ^a^	6 (3–6) ^b^
AS	7 (2–10) ^c^	8 (2–10) ^d^
BW (g)	350 (217–490) ^c^	270 (120–370) ^d^
BT (°C)	33.3 (32.2–34.7)	33.5 (32.2–34.5)

^a,b^ denotes significant differences within rows, with *p* < 0.05; ^c,d^ denotes significant differences within rows, with *p* < 0.001.

**Table 10:** Distribution of the sub-scores for Grimace expressed as the number and (%) for the 89 EB and FB puppies alive at 7 d of age.

	Grimace		
	Sub-Score 0 n (%)	Sub-Score 1 n (%)	Sub-Score 2 n (%)
EB (n = 39)	11 (28.2) ^a^	21 (53.9)	7 (17.9) ^c^
FB (n = 50)	1 (2) ^b^	20 (40)	29 (58) ^d^
TOTAL (n = 89)	12 (13.5)	41 (46.1)	36 (40.4)

^a,b^ denotes significant differences within columns, with *p* < 0.05; ^c,d^ denotes significant differences within columns, with *p* < 0.01.

## Data Availability

The data presented in this study are available on request from the corresponding author.
